# Increased neutrophil extracellular traps formation in the bronchoalveolar lavage fluid of dogs with bronchiectasis

**DOI:** 10.3389/fvets.2026.1786801

**Published:** 2026-03-18

**Authors:** Aurélie Lyssens, Pierre Janssen, Cécile Clercx, Thomas Marichal, Coraline Radermecker, Frédéric Billen

**Affiliations:** 1 Department of Clinical Sciences, Faculty of Veterinary Medicine, University of Liege, Liège, Belgium; 2 Laboratory of Immunophysiology, GIGA Institute, Liège University, Liège, Belgium

**Keywords:** bronchi, canine, immunity, inflammation, neutrophil extracellular traps, respiratory

## Abstract

Chronic bronchial diseases (CBD) associated with bronchiectasis (BE) and/or bronchomalacia (BM) are commonly diagnosed in dogs. Their pathogenesis remains incompletely understood, and recent studies have questioned the clinical relevance of distinguishing BE and/or BM, suggesting that their pathogenic mechanisms may overlap and that differentiating between them may have limited clinical significance. To date, the role of neutrophil extracellular traps (NETs) in BE and/or BM remains undetermined. This study aimed to validate NETs detection techniques in banked canine bronchoalveolar lavage fluid (BALF) using healthy (H) dogs, as negative controls, dogs with bacterial bronchopneumonia (BBP), as positive controls, and to compare NET detectability in dogs with CBD associated with BE and/or BM. A retrospective observational study was conducted using client-owned dogs presented with CBD and BE, BM, or BEBM based on endoscopic findings, or BBP. NETs were measured in BALF by quantification of cell-free DNA (cfDNA), detection of myeloperoxidase (MPO)-DNA complexes via enzyme-linked immunosorbent assay (ELISA) and by confocal laser scanning microscopy of immunofluorescent labeled samples. cfDNA concentrations were significantly higher in BBP dogs than in CBD or H-dogs, with no significant differences among the CBD groups. MPO–DNA complex levels were significantly higher in dogs with CBD and BBP than in H-dogs, with BE dogs showing the greatest increase compared with H and BM dogs, and BEBM dogs also exceeding H-dogs. Across all dogs, MPO–DNA complexes correlated positively with total and absolute neutrophil counts, neutrophil percentage, cfDNA concentrations, and duration of clinical signs. These associations varied between groups, with the strongest and most consistent correlations observed in BE-dogs. This study indicates that NETs are detected and quantifiable in the BALF of dogs with CBD and BE and/or BM and dogs with BBP. Their presence suggests that NET formation may play a role in the pathogenesis of CBD, especially in cases involving BE. Further research is needed to explore the role of NETs as well as the therapeutic potential of NET modulation in respiratory diseases.

## Introduction

Chronic bronchitis (CB), frequently diagnosed in both human and veterinary medicine, is characterized by chronic inflammation and excessive mucus production (1). In dogs, such persistent inflammation can lead to degenerative bronchial changes, including bronchiectasis (BE) and bronchomalacia (BM), or both (BEBM) (2–5). The exact pathogenesis of BE and/or BM is not fully understood, although morphological differences have been identified between BE and BM using various imaging modalities, such as thoracic radiography, computed tomography, and bronchoscopy (2, 4), only limited clinical and pathological differences between dogs with BE and/or BM have been found (5), raising questions about the clinical relevance of distinguishing these entities. This suggests that the underlying pathogenic mechanisms may potentially overlap, while factors leading to one or another of these specific conditions remain yet to be discovered.

Since neutrophilic inflammation is commonly observed in CB, the role of neutrophil extracellular traps (NETs) is gaining more and more attention in human medicine (1, 6, 7). NETs are web-like structures, composed of DNA fibers coupled to citrullinated histone 3 (cit-H3), and antimicrobial proteins such as myeloperoxidase (MPO), neutrophilic elastase (NE), cathepsin G, LL37 and defensins, that entrap bacteria, viruses, protozoa and fungi (8–11). Hereby, pathogens are immobilized and exposed to a locally high and lethal concentration of antimicrobial proteins (7, 9–12). However, excessive NETs production or inadequate NETs clearance may be harmful and induce deleterious effects on the host tissue (11, 13, 14). In human medicine, it has been shown that NETs formation plays an important role in the pathogenesis of many chronic neutrophilic lung diseases such as asthma, cystic fibrosis and, more recently, BE (6, 7).

The role of NETs in BM and in bacterial bronchopneumonia (BBP) remains unclear. Studies in murine models of BBP have demonstrated NETs formation, particularly in infections that cause significant lung injury and necrosis, such as *Klebsiella pneumoniae*, *Streptococcus pneumoniae*, and *Escherichia coli* (15). Additionally, research on humans with ventilator-associated pneumonia has shown increased NETs in the alveolar spaces (16). These data indicate that acute bacterial infection of the lower airways is a potent natural trigger of NETs release. Limited literature is available on NETs formation in BM, likely because this condition rarely occurs in humans. In veterinary medicine, NETs have been described in blood, tracheal wash fluid, and effusion samples, but exclusively in the context of systemic, infectious, or non-respiratory diseases, including sepsis, immune-mediated hemolytic anemia, canine leishmaniosis, Trypanosoma cruzi infection, trauma, and heat stroke, as well as in cats with FeLV infection and in horses with asthma (9, 11, 17–23). These studies consistently reported increased NETs markers, supporting the involvement of NETs. However, various methods were used across these studies to detect NETs. Each of the commonly used methods for NETs detection have important limitations: quantification of cell-free DNA is sensitive but nonspecific; MPO–DNA ELISAs provide a more specific yet indirect measurement; and immunofluorescent co-localization of markers such as cit-H3 and MPO enables direct visualization but remains low-throughput and labor-intensive (9, 11, 23). Despite these advances, NETs formation has never been investigated in canine respiratory disease, and no study has assessed their presence in the bronchoalveolar lavage fluid (BALF).

This study aimed to validate a technique previously applied in human and equine medicine for the detection and assessment of NETs in BALF samples from dogs. Healthy (H) dogs were included as a negative control group, and dogs with BBP served as a positive control group. The inclusion of BBP dogs as a positive control was based on evidence from human and experimental animal studies showing that acute bacterial infection of the lower airways elicits a NET response, making BBP a biologically justified model for NET-positive pulmonary inflammation (15, 16). After validation, the technique was applied to dogs with CBD associated with BE and/or BM to compare NETs detectability among groups. We hypothesized that NETs can be detected in the BALF of H-dogs, that their levels are increased in BBP-dogs, and that significant differences may exist between BE- and/or BM- and H-dogs, as well as between H- and BBP-dogs.

## Materials and methods

### Study population

H-dogs included clinically healthy client-owned dogs and healthy experimental Beagle dogs. All individuals had no clinical signs of disease, showed normal findings on physical examination, haematology, and serum biochemistry, and displayed a normal gross bronchoscopic appearance with no abnormalities detected on BALF analysis. Samples from healthy client-owned dogs and experimental Beagles were obtained from previously collected and stored material, originally gathered under approved experimental protocols (Ethical Committee of the University of Liège; protocol #1435 and #2299, respectively) and with informed owner consent where applicable.

BALF samples of client-owned dogs diagnosed with acute BBP at the Small Animal Teaching Hospital, Liège, Belgium, but without concurrent BE and/or BM, were also retrospectively included. The diagnosis of BBP was based on compatible clinical and physical examination findings, characteristic abnormalities on thoracic radiographs (focal, multifocal, or diffuse alveolar patterns), bronchoscopy findings, and BALF analysis showing septic inflammation, combined with a positive bacterial culture and/or quantitative polymerase chain reactions (qPCR) result (24).

Lastly, client-owned dogs, presented at the same institution for chronic cough between February 2015 and October 2022 and diagnosed with CBD with BE, BM or BEBM based on endoscopy findings according to previously described criteria, were retrospectively included in the study (5). Briefly, BE was defined as an obvious macroscopic lack of tapering or increased diameter of the bronchial lumen and BM was defined as dynamic airway collapse of the bronchi whereby changes in luminal diameter of at least 25% were observed during respiration.

Data collected from the medical records included age, sex, breed, body weight, duration of symptoms, previous or ongoing treatment, results of total cell count (TCC), differentiated cell count (DCC) and absolute neutrophil count (ANC) on BALF, results of traditional semi-quantitative bacterial culture and qPCR results for *Bordetella bronchiseptica* and *Mycoplasma cynos* on BALF. Normal TCC was considered less than 600 cells per microliter, and neutrophilic inflammation was concluded if >12% of neutrophils were observed (25). The ANC in BALF was calculated by multiplying the TCC by the percentage of neutrophils obtained from the DCC. Aerobic culture testing was performed by a commercial veterinary diagnostic laboratory (Antech, Liège, Belgium) and qPCR testing for *Bordetella bronchiseptica* and *Mycoplasma cynos* was performed at the clinical pathology laboratory (Department of Veterinary Pathology, Liège, Belgium). Infection was defined as the combined presence of an increased TCC, presence of neutrophilic inflammation, presence of intra- or extracellular bacteria and positive culture [>1.7 × 10^3^ colony-forming units (CFU) per millilitre of BALF] and/or positive qPCR results [cycle threshold (Ct) < 34] (26, 27). Dogs that tested positive for *Bordetella bronchiseptica* were excluded for further NET analysis since it has been shown that *Bordetella pertussis* inhibits NET formation by suppressing the oxidative burst necessary for NETosis (28).

For all dogs with CBD and BE and/or BM based on endoscopy, a final diagnosis was also attributed based on all available data. Dogs were diagnosed with eosinophilic bronchopneumopathy (EBP) if they exhibited a moderate to severe broncho-interstitial pattern, abundant yellow-green mucopurulent material on bronchoscopy, and more than 50% eosinophils in the BALF (29). A diagnosis of parasitic bronchitis was made when parasites were observed either during bronchoscopy, identified in BALF analysis by qPCR, or detected through a Baermann test on faeces. Dogs were suspected to have idiopathic pulmonary fibrosis (CIPF) based on the signalment, clinical signs at presentation, compatible CT findings, exclusion of other cardiopulmonary diseases, and histopathology of the lungs, if available (30). Lastly, dogs were diagnosed with CB based on history of coughing for more than 2 months, an increased TCC and non-septic neutrophilic inflammation on BALF analysis, and no other specific underlying cause (1).

### BALF sample collection and processing

BALFs were obtained in each dog under anaesthesia as previously described (31). Dogs were anesthetized without intubation. The paediatric bronchoscope was cleaned and disinfected using an endoscopy washing machine (Serie 4-PA, Soluscope®, France) before each use. The bronchoscope was then inserted into the trachea, taking care to avoid contact with the oral and tracheal mucosa. The BAL was performed by injecting 3–4 mL/kg of sterile saline solution divided into 3 aliquots, including 2 aliquots with the endoscope inserted into the right diaphragmatic lobe, followed by a 3rd aliquot placed into the left diaphragmatic lobe. Each aliquot was directly aspirated by gentle suction and the fluids recovered from the 3 aliquots were pooled. A small amount of naïve BALF was directly used for calculation of the TCC as well as for the cytospin preparation. For the latter, 150 μL of BALF was added to a cytospin funnel, which was subsequently centrifuged at 1000 rpm (112 × *g*) for 4 min using a rotor JC 301 Cellspin I 1206-14 (Tharmac). Cytospin preparations were stained by Diff Quick and were used for differential cell count (DCC) determination by counting a minimum of 100 cells. After centrifugation of BALF, supernatant of BALF were transferred into cryotubes and stored at −80 °C until analysis.

### Low specificity quantification of cfDNA in BALF supernatant

This method was applied in this study to verify the presence of extracellular DNA in the BALF supernatant (32, 33). It is a highly sensitive method, allowing rapid and quantitative detection of low concentrations of double-stranded DNA, and is relatively cost-effective (32–36). However, it does not provide information on the origin of the detected DNA and therefore cannot discriminate between DNA released through NETs formation, apoptosis, or other cellular processes (37, 38).

To quantify the concentration of cfDNA in the supernatant, the Quant-iT PicoGreen dsDNA Assay Kit (Invitrogen, Carlsbad, CA, P7589) was used following the manufacturer’s protocol. A standard curve ranging from 0.4 μg/mL to 3.125 × 10–3 μg/mL was prepared to estimate the double-stranded (ds)DNA concentration in the BALF samples. Samples from dogs in the H, BE, BM, and BEBM groups were diluted 1:5, while samples from dogs with BBP were diluted 1:10 for the quantification of cfDNA. Quant-iT PicoGreen reagent was added directly to the wells. Fluorescence signals were measured using an Infinite 200 PRO multimode plate reader (Tecan Group Ltd., Switzerland), with filter settings of 485 nm and 535 nm.

### ELISA detecting MPO–DNA complexes in BALF supernatant

To confirm the presence of NETs and provide a more specific assessment than cfDNA quantification alone, an adapted sandwich ELISA was performed in duplicate to detect MPO–DNA complexes, as previously described (9, 11). MPO–DNA complexes are considered more specific indicators of NET formation than either DNA or MPO alone, as extracellular DNA may originate from any dying cell, and MPO is present in neutrophils regardless of NET release (9, 11, 39). The simultaneous detection of both MPO and DNA within a complex therefore strongly suggests active NET formation (9, 11). For the assay, 96-well flat-bottom plates were coated overnight at 4 °C with goat anti-human/mouse MPO antibody (3.125 μg/mL, R&D Systems, AF3667) diluted in PBS. The following day, the plates were blocked for 1 h with PBS containing 1% bovine serum albumin (BSA, Sigma, A7906) to prevent non-specific binding. After blocking, the BALF samples were added to the wells. To enhance the accessibility of the MPO-DNA complexes for antibody binding and detection, the samples were treated with small amounts of DNase I (RNase-free, 125 U, Sigma, 11284932001) for 15 min, as previously described (9, 11). A DNase concentration of 125 U was chosen to maximize the detection of MPO-DNA complexes while preventing excessive degradation of the NETs, as previously reported for this ELISA method (9, 11). After the DNase treatment, the enzymatic reaction was stopped by adding 1 μL of PBS-EDTA (0.05 M), and the plates were incubated for 90 min at room temperature. Mouse anti-DNA detection antibodies (1.10–2 μg/mL, clone BV16-13, Sigma-Aldrich, MAB030) were then added and incubated for 1 h. Subsequently, biotinylated polyclonal rat anti-mouse IgG2a (1.10–2 μg/mL, BD Biosciences, 553388) was added and incubated for 90 min. Following these incubations, the plates were washed and streptavidin-conjugated horseradish peroxidase (HRP) (1:500 dilution, ThermoFisher, 18-4100-94) was added for a 30-min incubation. After washing the plates again, the substrate 3,3′,5,5’-Tetramethylbenzidine (TMB, Lifetechnologies, SB02) was added and incubated in the dark, allowing the enzymatic reaction to proceed. The reaction was then stopped by adding 1 M H2SO4. The absorbance was measured at 450 nm using a Multiskan FC plate reader (ThermoScientific, 51119000), and no background or reference wavelength correction was applied. Throughout the procedure, 3 to 5 washes were performed between each step using PBS-Tween-20 0.05% (ThermoFisher, 233360010) to ensure optimal signal clarity.

### Immunofluorescence staining and analysis of BALF

Following quantitative assessment of MPO–DNA complexes, immunofluorescence (IF) staining was performed to obtain morphological visualization of NETs within BALF samples (40). Immunofluorescence staining was performed on BALF cytospins from 1 dog with BE and 1 dog with BBP, as previously described (11). The cytospin slides were permeabilized with 2% Triton X-100 in PBS. To block non-specific binding, the samples were incubated with a blocking buffer consisting of PBS with 2% bovine serum albumin (BSA) and 2% fetal donkey serum (FBS; Sigma-Aldrich) for 1 h at room temperature. The samples were then stained with rabbit anti-human citrullinated histone H3 (Cit-H3, Abcam, Ab5103) at a 1:100 dilution and goat anti-human myeloperoxidase (MPO, R&D Systems, AF3667) at a 1:40 dilution in blocking buffer for 1 h at room temperature. Following incubation, the samples were washed with PBS, and secondary antibodies were applied: donkey anti-rabbit Alexa Fluor 568 (ThermoFisher, A10042, 1:200 dilution) and donkey anti-goat Alexa Fluor 488 (ThermoFisher, 11055, 1:200 dilution). These secondary antibodies were prepared in blocking buffer containing 4′,6-diamidino-2-phenylindole (DAPI, ThermoFisher, D3571, 1:1000 dilution) to stain the nuclei. The samples were incubated for 2 h in the dark at room temperature. After incubation, the samples were mounted with 10 μL of ProLong Antifade reagent (Thermo Fisher, P36961) on glass slides and stored in the dark at room temperature overnight. All samples were analysed using fluorescence microscopy with standard filter sets. For image acquisition, a Zeiss LSM 880 Airyscan Elyra S.1 confocal microscope (Zeiss) was used. Images were processed using ImageJ software. Negative and isotype control staining were not performed for the immunofluorescence analyses. However, the primary antibodies used have been previously validated and applied in our laboratory in studies involving murine, human, and equine samples, where appropriate specificity controls were included (11, 23, 41).

### Statistical analysis

Categorical variables were described using frequency tables (numbers and percentages) and continuous variables were summarized using mean ± standard deviation (SD) when normally distributed, median and quartiles (Q1, Q3) and minimum and maximum values. Characteristics of groups were compared using chi-square test (or Fisher exact test) for categorical variables and ANOVA (or Kruskall–Wallis for asymmetric variables) for continuous variables. Symmetric variables were described by mean ± SD and asymmetric variables were described by median (Q1; Q3). Pairwise comparisons were performed when significant differences are detected. To explore the relationships between MPO–DNA complex levels and clinical or cytological variables, including age, body weight, duration of clinical signs, TCC, ANC, and the percentages of macrophages, neutrophils, eosinophils, and lymphocytes in BALF, as well as cfDNA concentrations in BALF, Spearman correlation coefficients were calculated across all dogs and within diagnostic groups. Significant level was set at 5% (*p* < 0.05). Calculations were conducted using SAS software (version 9.4) and graphics were produced using R software (version 4.4.1). No power calculation was performed, as this was a retrospective exploratory study based on the availability of archived bronchoalveolar lavage fluid samples.

## Results

### Study population and BALF cell analysis

A total of 53 dogs was included in the study and categorized into 5 groups according to the diagnosis: BE, BM, BEBM, BBP and H. Groups characteristics are reported in Table 1. No statistically significant differences in weight and sex were observed among the 5 groups. Dogs diagnosed with BEBM were significantly older than those with BBP (*p* = 0.025). The duration of clinical signs also differed among diagnostic groups, with a longer clinical course observed in dogs with BE and/or BM compared to those with BBP (*p* < 0.001).

**Table 1:** Characteristics of the dogs according to the endoscopic diagnosis and the results of bacterial culture and quantitative polymerase chain reaction results.

Variable	BE	BM	BEBM	BBP	H
*N*	9	11	12	6	15
Sex (M/F)	4/5	5/6	7/5	3/3	7/8
Age (year)	9.22 ± 2.73	9.09 ± 2.81	10.17 ± 2.55	5.67 ± 3.33	8.07 ± 2.55
Weight (kg)	13.97 ± 9.42	10.94 ± 9.19	15.01 ± 5.96	24.28 ± 10.73	17.61 ± 9.05
Duration of clinical signs (months)	9.00 (3.00;12.00)	8.00 (4.00;24.00)	8.00 (6.00;24.00)	1.00 (0.50;1.00)	0.00 (0.00;0.00)
Years of BALF sample collection	2016–2021	2015–2021	2015–2021	2015–2022	2012–2021
Breeds	3 crossbreed, 2 Dachshund, 1 Beagle, 1 Fox Terrier, 1 Malinois, 1 Maltese	3 crossbreeds, 2 Sheltie, 1 Poodle, 1 Labrador retriever, 1 Yorkshire Terrier, 1 Jack Russel Terrier, 1 WHWT, 1 Pomerian	4 WHWT, 3 crossbreeds, 1 Whippet, 1 Munsterlander, 1 Samoyed, 1 Basset Fauve, 1 Beagle	1 American Staffordshire terrier, 1 Bouvier de Flandres, 1 American Pitbull, 1 Griffon Bruxellois, 1 Australian shepherd, 1 Malinois	4 Beagle, 2 Border collie, 1 Jack Russel Terrier, 1 American Staffordshire Terrier, 1 German Shepherd, 1 Dobermann Pinscher, 1 Malinois, 1 Yorkshire Terrier, 1 CKCS, 1 Labrador retriever, 1 crossbreed
Final diagnosis	8 chronic bronchitis, 1 EBP	10 chronic bronchitis, 1 CIPF	8 chronic bronchitis, 3 CIPF, 1 EBP	5 not specified, 1 suspicion of foreign body in lung lobe	/

Data are expressed as mean ± standard deviation regarding “age” and “weight” and expressed as median and interquartile range regarding duration of clinical signs.

BE, bronchiectasis; BM, bronchomalacia; BEBM, combination of bronchiectasis and bronchomalacia; BBP, bacterial bronchopneumonia; H, healthy; M, male; F, female; BALF, bronchoalveolar lavage fluid; WHWT, West Highland White Terrier; CKCS, Cavalier King Charles Spaniel; EBP, eosinophilic bronchopneumopathy; CIPF, canine idiopathic pulmonary fibrosis.

Group characteristics regarding BALF cell analysis are reported in Table 2. TCC was markedly higher in BBP-dogs compared to dogs with BE, BM, and BEBM, and all disease groups showed higher TCCs than H-dogs (*p* < 0.0001). The DCC also differed significantly among groups. Neutrophil percentage in BALF was significantly higher in BBP compared with BM and H-dogs (*p* = 0.0023) and macrophage percentage in BALF was significantly lower in BBP than in BM and H-dogs (*p* = 0.0011). Lymphocyte percentage in BALF was significantly lower in BBP than in H-dogs (*p* = 0.023). No significant differences were observed in eosinophil percentage among the 5 groups. ANC was significantly higher in BBP compared to BE, BM, BEBM, and H-dogs (*p* < 0.0001), while ANC in H-dogs was lower than in BEBM (*p* = 0.001).

**Table 2:** Characteristics of the total and differential cell count in bronchoalveolar lavage fluid of dogs in each diagnostic group.

Group	TCC (cells/μl)	Macrophages (%)	Neutrophils (%)	Lymphocytes (%)	Eosinophils (%)	ANC (cells/μl)
BE	1,024 (720–1,320)	50.22 ± 31.66	16.00 (9.00–65.00)	2.00 (0.00–5.00)	2.00 (0.00–4.00)	152 (64.8–665.6)
BM	800 (340–2,000)	63.64 ± 27.19	17.00 (9.00–40.00)	4.00 (1.00–5.00)	0.00 (0.00–2.00)	417.6 (19.8–720.8)
BEBM	1,620 (900–2,315)	52.00 ± 29.87	21.50 (8.00–47.50)	1.50 (0.00–11.00)	3.00 (0.00–12.00)	246.5 (73.9–987)
BBP	9,750 (2,700–24,475)	20.67 ± 20.96	85.00 (58.00–95.00)	1.00 (0.00–2.00)	0.00 (0.00–0.00)	9267.5 (1,215–22,950)
H	320 (250–390)	74.30 ± 14.10	9.00 (6.00–15.00)	11.00 (5.00–23.00)	2.00 (0.50–3.00)	25.2 (18.9–48)

Data are expressed as median and interquartile range for TCC, ANC, % neutrophils, % lymphocytes and % eosinophils. Data are expressed as mean ± standard deviation for % macrophages.

TCC, Total cell count per μl; ANC, absolute neutrophil count; BE, bronchiectasis, BM, bronchomalacia, BEBM, combination of bronchiectasis and bronchomalacia; BBP, bacterial bronchopneumonia; H, healthy.

Of the six dogs with BBP, semi-quantitative bacterial culture identified *Pasteurella canis* in two dogs, *Staphylococcus pseudintermedius* in two dogs, *Pseudomonas aeruginosa* in one dog for and *Streptococcus equi* subsp*. zooepidemicus* in one dog. Bacterial culture and qPCR analysis was negative in all BE and/or BM and H-dogs.

### Low specificity quantification of cell-free DNA in BALF

**Figure 1:**
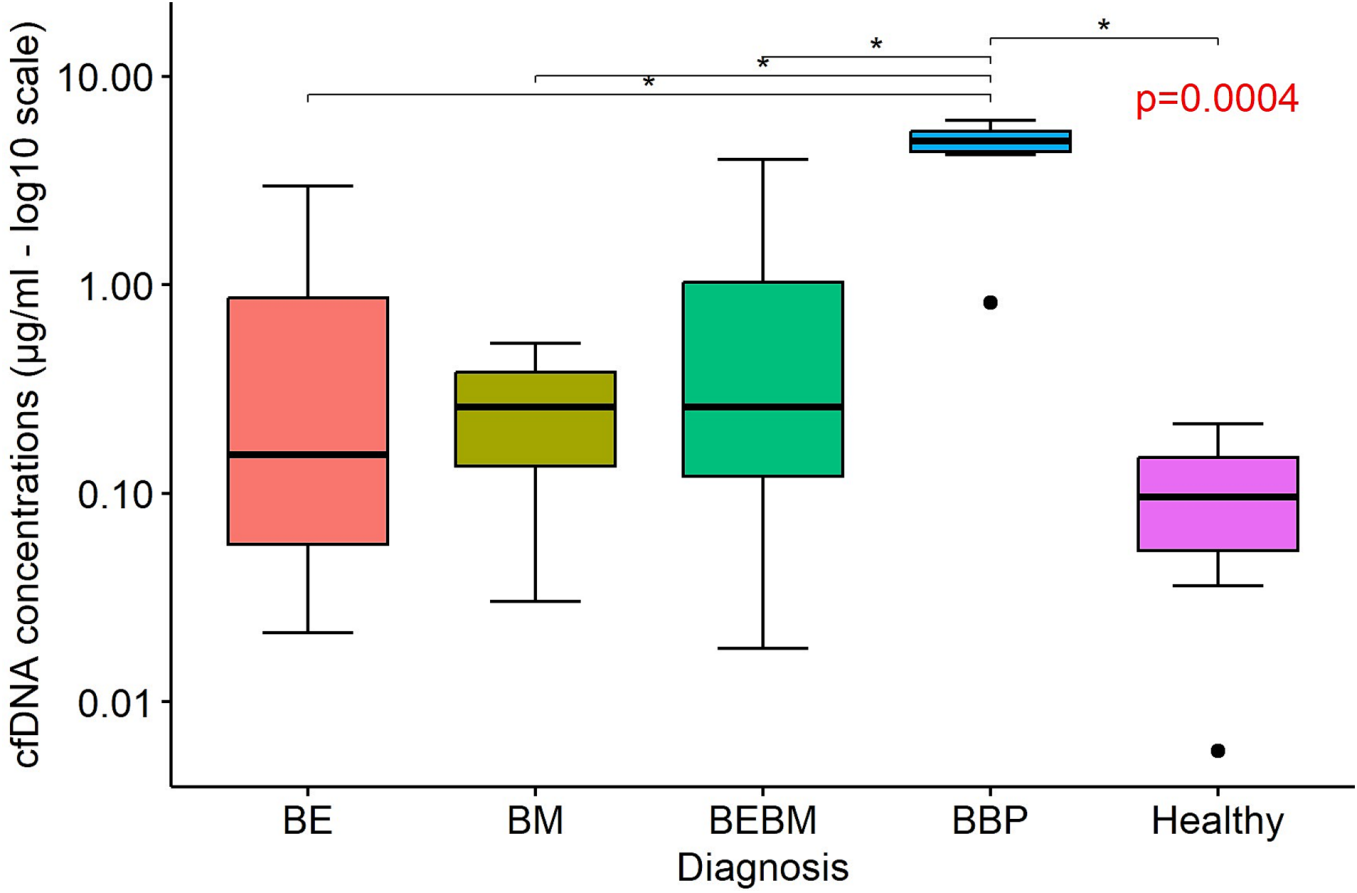
Boxplots illustrating the concentration of cell-free DNA (cfDNA; μg/ml) in bronchoalveolar lavage fluid supernatants from dogs with bronchiectasis (BE), bronchomalacia (BM), a combination of both conditions (BEBM), bacterial bronchopneumonia (BBP), and healthy control dogs. Each box represents the interquartile range (IQR: 25th–75th percentile), with the horizontal line indicating the median cfDNA concentration. Outliers are shown as individual dots. Logarithmic scaling (log_10_) was applied to the y-axis. CFDNA concentrations were significantly higher in dogs with BBP compared to healthy dogs and to those with chronic airway disease (BE and/or BM).

Low-specificity quantification of cfDNA in BALF was performed on 51 dogs (8 dogs with BE, 11 dogs with BM, 11 dogs with BEBM, 6 BBP-dogs and 15 H-dogs). cfDNA concentrations in BALF were significantly higher in dogs with BBP compared to H-controls (*p* = 0.0004). cfDNA concentrations of BBP-dogs were also significantly greater than those observed in dogs with CBD (*p* < 0.05 for all pairwise comparisons). No significant differences were observed among the CBD groups (BE, BM, BEBM) or between these and H-controls (Figure 1).

### ELISA detecting MPO–DNA complexes

An ELISA was performed on a cohort of 47 dogs (9 dogs with BE, 11 dogs with BM, 11 dogs with BEBM, 6 BBP-dogs and 10 H-dogs). The intra-assay coefficient of variation, calculated across all samples combined, was 5.75%. All samples were analysed in duplicate on a single 96-well plate during one assay run; therefore, inter-assay variability was not applicable and only intra-assay coefficient of variation was calculated.

**Figure 2:**
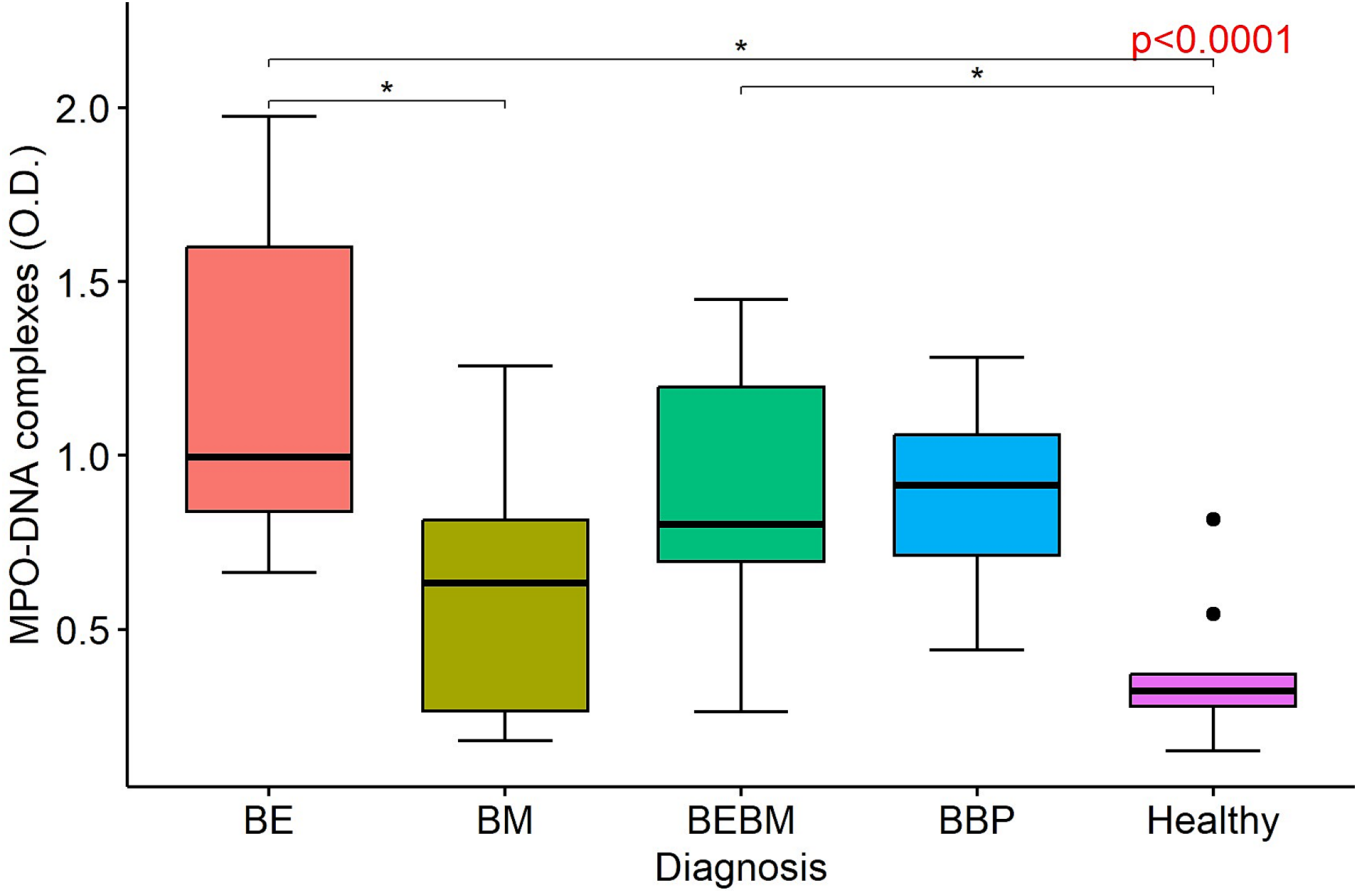
Boxplots illustrating the concentration of myeloperoxidase (MPO)–DNA complexes, expressed as optical density (O.D.), in bronchoalveolar lavage fluid from dogs with bronchiectasis (BE), bronchomalacia (BM), a combination of both conditions (BEBM), bacterial bronchopneumonia (BBP), and healthy control dogs. Each box represents the interquartile range (IQR: 25th–75th percentile), and the horizontal line within the box indicates the median value. Outliers are displayed as individual dots.

MPO–DNA complex levels, expressed as optical density, were overall significantly higher in dogs with CBD and BBP compared with H-dogs (*p* < 0.0001). Pairwise analysis showed higher levels of MPO–DNA concentrations in BE-dogs compared with H-dogs (*p* < 0.05) and BM-dogs (*p* < 0.05), while BEBM-dogs also had higher levels of MPO–DNA complexes in their BALF compared to H-dogs (*p* < 0.05) (Figure 2). No statistically significant difference was found between BBP-dogs and dogs with CBD.

### Immunofluorescence staining

IF staining was processed in one dog with BE (Supplementary Figure S1) and in one dog with BBP (Supplementary Figure S2). In both slides, extracellular web-like structures were observed that stained positive for MPO and cit-H3, consistent with the presence of NETs. The extracellular DNA backbone of these structures was counterstained with 4′, 6-diamidino-2-phenylindole (DAPI). These structures were observed in close proximity to neutrophils and appeared as diffuse, filamentous material extending into the extracellular space, suggestive of neutrophil decondensation and chromatin release.

### Association between MPO–DNA complexes and other measures

**Figure 3:**
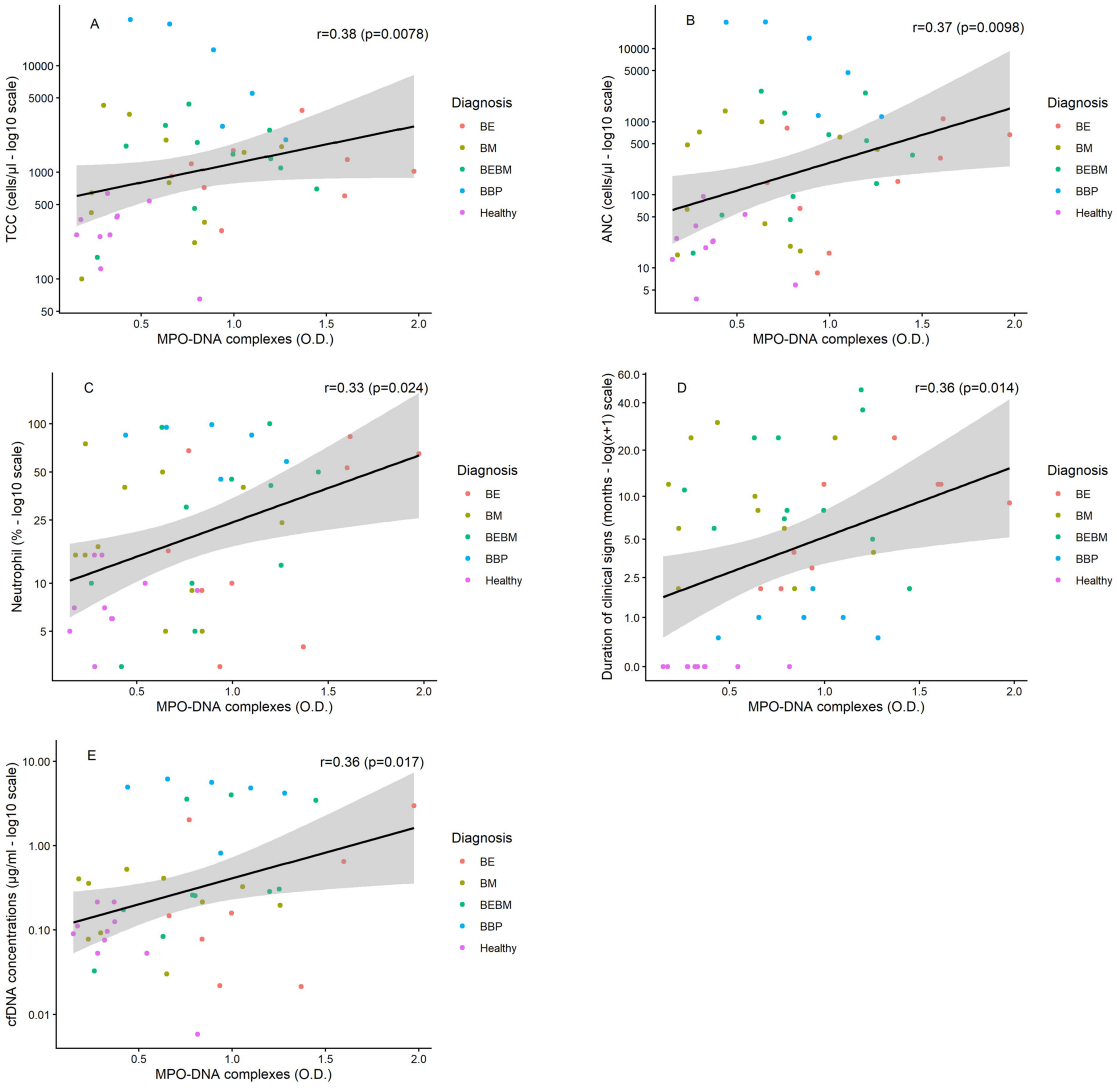
Scatterplots illustrating the associations between MPO–DNA complexes (expressed as optical density, O.D.) and selected clinical or cytological variables, including total cell count (TCC), absolute neutrophil count (ANC), percentage of neutrophils in BALF, duration of clinical signs, and cfDNA concentrations (**A–E**, respectively). Each point represents one dog and is color-coded by diagnosis. The solid black line represents the global linear association across all dogs, with the shaded area indicating the 95% confidence interval. Logarithmic scaling (log_10_) was applied to *y*-axes for TCC, ANC, neutrophil percentage and cfDNA concentrations. BE, bronchiectasis; BM, bronchomalacia; BEBM, combined bronchiectasis and bronchomalacia; BBP, bacterial bronchopneumonia.

Across all dogs, MPO–DNA complexes were positively correlated with TCC (rs = 0.38; *p* = 0.0078), ANC (rs = 0.37; *p* = 0.0098), neutrophil percentage (rs = 0.33; *p* = 0.024), cfDNA concentrations (rs = 0.36; *p* = 0.017), and duration of clinical signs (rs = 0.36; *p* = 0.014), and negatively correlated with macrophage percentage (rs = −0.40; *p* = 0.0052) (Supplementary Table 1; Figures 3A–E). However, within diagnostic subgroups, several correlations differed in direction or lost significance. In particular, the positive global association between MPO–DNA complexes and TCC or ANC was reversed in dogs with BBP (TCC: rs = −0.94; *p* = 0.0048; ANC: rs = −0.89; *p* = 0.019), whereas cfDNA concentrations remained positively and significantly associated with MPO–DNA complexes only in dogs with BEBM (rs = 0.68; *p* = 0.029). In contrast, in dogs with BE, MPO–DNA complexes showed weak to moderate positive correlations with selected variables, with all Spearmann correlation coefficients remaining below 0.5, although they did not consistently reach statistical significance (Table 3). Additionally, in dogs with BE, MPO–DNA complexes were positively correlated with duration of clinical signs (rs = 0.72; *p* = 0.028), a relationship not observed in the other diagnostic groups (Table 3; Figure 3D).

**Table 3:** Spearman correlation coefficients between MPO–DNA complexes and other variables in dogs stratified by endoscopic diagnosis.

Variable	BE		BM		BEBM		BBP		H	
	Correlation	*p*-value	Correlation	*p*-value	Correlation	*p*-value	Correlation	*p*-value	Correlation	*p*-value
Age (years)	−0.05	0.90	0.30	0.37	−0.33	0.33	0.09	0.87	−0.02	0.96
Weight (kg)	−0.13	0.73	0.44	0.17	0.33	0.33	−0.43	0.40	0.33	0.35
Duration of clinical signs (months)	0.72	0.028*	−0.16	0.65	−0.21	0.54	0.06	0.91	/	/
TCC (cells/μl)	0.22	0.58	0.17	0.61	−0.15	0.65	−0.94	0.0048*	0.15	0.69
Macrophage (%)	−0.35	0.36	−0.13	0.70	−0.39	0.23	0.37	0.47	−0.05	0.88
Neutrophil (%)	0.30	0.43	−0.12	0.73	0.45	0.17	−0.49	0.32	0.22	0.54
Lymphocyte (%)	0.48	0.18	0.51	0.11	−0.32	0.33	0.83	0.039*	−0.01	0.99
Eosinophil (%)	0.03	0.93	−0.10	0.77	0.56	0.076	0.39	0.44	0.09	0.81
ANC (cells/μl)	0.42	0.26	0.03	0.94	0.26	0.43	−0.89	0.019*	0.01	0.99
cfDNA (μg/ml)	0.24	0.57	−0.16	0.63	0.68	0.029*	−0.66	0.16	−0.37	0.29

*Indicates a statistically significant correlation.

TCC, Total cell count per μl; ANC, absolute neutrophil count; cfDNA, cell-free DNA; BE, bronchiectasis, BM, bronchomalacia, BEBM, combination of bronchiectasis and bronchomalacia; BBP, bacterial bronchopneumonia; H, healthy.

## Discussion

To the author’s knowledge, this study is the first to demonstrate the presence and quantifiable detection of NETs in the BALF of healthy dogs, dogs with BBP and dogs with CBD (BE and/or BM). By combining low-specificity cfDNA quantification, ELISA for detection of MPO-DNA complexes, and in a limited way, IF staining, we provide evidence that NETs are present in the airways of canine inflammatory bronchopulmonary diseases.

Although NETs have previously been described in veterinary medicine across various diseases contexts and biological fluids, as outlined in the introduction, direct comparison with those studies is limited. This is largely due to the heterogeneity of NET detection methods employed, including cfDNA quantification, ELISA-based assays, and IF, which differ in specificity and interpretability and complicates meaningful quantitative comparison across studies and sample types.

Dogs diagnosed with CBD (BE, BM, BEBM) and BBP exhibited significantly higher concentrations of MPO-DNA complexes in BALF compared with healthy controls, confirming NETs presence in diseased airways. MPO–DNA detection is considered a specific indicator of NETs formation, as it reflects the biochemical coupling of MPO and DNA during chromatin decondensation and extrusion, unlike cfDNA quantification alone, which lacks cellular specificity (8, 9, 11).

Across all dogs, MPO–DNA complexes correlated positively with TCC, ANC and neutrophil percentage. These associations indicate that NETs formation parallels the degree and presence of neutrophilic inflammation. However, subgroup analyses revealed disease-specific differences. Notably, dogs with BE in particular showed the highest amount of MPO-DNA complexes compared to BM and H-dogs, suggesting a potential greater contribution of NETs to the pathophysiology of BE. These findings are consistent with observations in human medicine, where elevated NETs markers have been detected in the sputum and BALF of patients with BE and other chronic neutrophilic lung diseases (42, 43). In the BE-group, the high amount of MPO-DNA complexes and the lack of a significant positive correlation between MPO-DNA complexes and either neutrophil percentages or ANC indicates that the elevation of MPO–DNA in BE is not simply a consequence of increased neutrophil numbers, but may rather reflect heightened neutrophil activation and NETosis. In other words, NETs formation in BE appears to be driven by qualitative changes in neutrophil behaviour rather than by the quantitative presence of these cells. This observation may support the hypothesis that chronic airway injury in dogs with BE might involve dysregulated neutrophil function, as described for human BE and chronic neutrophilic airway disease, leading to increased NETs release even in the absence of proportionally greater neutrophil infiltration (7, 13, 43). Interestingly, dogs classified within the BEBM group also showed elevated MPO–DNA complex levels compared to healthy controls. This likely reflects the contribution of BE changes within this subgroup, as BE was associated with the highest degree of neutrophil activation and NETs formation among the chronic respiratory conditions evaluated.

The positive correlation observed between MPO–DNA complexes and the duration of clinical signs, specifically in dogs with BE, may further supports the role of persistent neutrophil activation and NETs formation in the pathophysiology of chronic airway inflammation in this subgroup. Prolonged clinical disease likely reflects chronic epithelial injury and ongoing inflammatory stimulation, which may result in persistent or cumulative NETs presence within the airways (7). Such chronic NETs accumulation may perpetuate inflammation by releasing cytotoxic and proinflammatory components, thereby promoting tissue remodelling and airway damage (7, 44, 45).

Although MPO-DNA complexes differed significantly between CBD and healthy dogs, cfDNA concentrations did not differ significantly between groups. This discrepancy suggests that cfDNA quantification alone may not specifically reflect NET-associated DNA, as it can also originate from other forms of cell death such as apoptosis, necrosis or tissue injury (11, 36). In this context, dogs with BBP exhibited the highest cfDNA concentrations among all groups but their MPO–DNA complex levels were low. Spearman correlation coefficient analysis revealed that, in contrast to the global cohort, BBP dogs showed negative correlations between MPO–DNA complexes and TCC and ANC, and no significant association with neutrophil percentage. Together, these findings suggest that the elevated cfDNA in BBP primarily reflects extensive tissue damage and necrotic cell death due to the active inflammation, rather than ongoing NETs formation. The low MPO–DNA complex levels observed in BBP may reflect a predominance of neutrophil phagocytosis rather than NETs formation during bacterial infection (46, 47). In certain bacterial infections, neutrophils initially rely on phagocytosis, and NETs formation is not immediate (46, 47). For example, in the case of *S. aureus* (46), demonstrated that during the early minutes to hours of interaction, phagocytosis predominates. *S. aureus* triggered NETs formation only after an extended period *in vitro*, and within the first 60 min, neutrophils mainly killed *S. aureus* through phagocytosis rather than NETs release (46, 47). Low MPO–DNA levels in BBP may also result from neutrophil exhaustion or previous NETs release during the acute phase of infection, as well as subsequent degradation of NETs by proteolytic enzymes and nucleases in the inflamed lung (48). In addition, active bacterial evasion strategies may further contribute to the relatively low MPO–DNA levels observed in BBP, as degradation or suppression of NET formation would directly reduce measurable MPO–DNA complexes despite ongoing neutrophilic inflammation. Although NETs effectively ensnare pathogens, many microorganisms have developed mechanisms to evade this defence. Among the species identified in this study, *Staphylococcus pseudintermedius* has been shown to degrade NETs through the release of a thermostable nuclease (NucB), while *Pseudomonas aeruginosa* exhibits multiple strategies to resist NET-mediated killing, including suppression of NET formation and development of resistant phenotypes in chronic airway disease (48, 49). In addition, *Streptococcus equi* subsp*. zooepidemicus* produces extracellular nucleases capable of degrading the DNA backbone of NETs (50). In contrast, NET-evasion mechanisms have not been described for *Pasteurella canis* to date.

Several limitations should be considered when interpreting our findings. First, due to its retrospective nature, the study relied on previous collected clinical data and BALF samples. This may have introduced variability in sample handling, and sample storage. Although BALF supernatants were stored at −80 °C to minimize DNase activity, bacterial growth, and degradation, the time between collection and freezing was not standardized, which could affect cfDNA concentrations through evaporation or oxidative or hydrolytic changes. Moreover, the retrospective design of this study did not allow us to perform simultaneous measurement of cfDNA and MPO-DNA complexes in all samples. Secondly, BALF was collected at a single time point per dog, limiting assessment of the dynamics of NETs formation during disease progression or in response to therapy. This is especially relevant in BBP, where neutrophil exhaustion or previous NETs release could affect measurable MPO–DNA complexes. Another limitation is the small sample size per group. The BBP group in particular was limited in size, which may reduce generalizability of subgroup analyses leading to potential increased risk of type I errors. Additionally, the low number of cytospin slides available for IF restricted direct visualization of NETs to only two samples (one BE and one BBP dog). Also, no quantification of the NETs based on IF could be performed. Moreover, IF analyses were performed without negative or isotope controls, which limits the ability to fully exclude non-specific staining. Although the antibodies used have been previously validated in our laboratory in other species with appropriate specificity controls, the absence of such controls in the present study limits definitive interpretation of the IF findings (11, 23, 41). Therefore, these findings should be interpreted as illustrative and supportive rather than confirmatory.

Future prospective studies should expand IF analysis to a larger cohort, include longitudinal sampling, and combine NETs quantification with IF imaging to better characterize NETs dynamics and their role in CBD.

Overall, the detection of NETs in BALF of canine BE and/or BM highlights a potential link between chronic neutrophilic inflammation and airway remodelling. These findings may provide new insights into the pathogenesis of CBD, suggesting that persistent NETs formation may contribute to airway damage. In conclusion, this study provides the first evidence of NETs in the lower airways of dogs with both chronic (BE and/or BM) and acute (BBP) bronchopulmonary diseases, reinforcing the translational parallels with human bronchial disorders and highlighting potential therapeutic strategies focused on NETs modulation (7, 13, 43).

## Data Availability

The raw data supporting the conclusions of this article will be made available by the authors, without undue reservation.

## Supplementary material

SUPPLEMENTARY FIGURE S1 Illustrative confocal microscopy pictures (objective 40x) of BALF neutrophils in bronchoalveolar lavage fluid of a dog with bronchiectasis **(A)**. Activated neutrophils showing extracellular myeloperoxidase (MPO; green; **B**)- and citrullinated histones 3 (Cit-H3; red; **C**)-positive structures, consistent with ongoing NETs formation, are indicated by purple arrows on **A**. The white arrows indicate presence of intracellular MPO and Cit-H3-positive structures on **A**. DAPI (4′,6-diamidino-2-phenylindole) is a fluorescent dye used to label DNA **(D)**. The scale bar indicates a length of 10 μm.

SUPPLEMENTARY FIGURE S2 Illustrative merged confocal microscopy pictures (objective 20x) of BALF neutrophils in bronchoalveolar lavage fluid of a dog with bacterial bronchopneumonia. NETs are defined as extracellular structures double positive for myeloperoxidase (MPO; green) and citrullinated histones (Cit-H3; red) and are indicated by white arrows. DAPI (4′,6-diamidino-2-phenylindole) is a fluorescent dye used to label DNA. The scale bar indicates a length of 50μm.
