# Supplementary figures and images for: Increased neutrophil extracellular traps formation in the bronchoalveolar lavage fluid of dogs with bronchiectasis
Source: Front Vet Sci. 2026 Mar 18;13:1786801. doi: 10.3389/fvets.2026.1786801 (PMC13040547; doi:10.3389/fvets.2026.1786801)

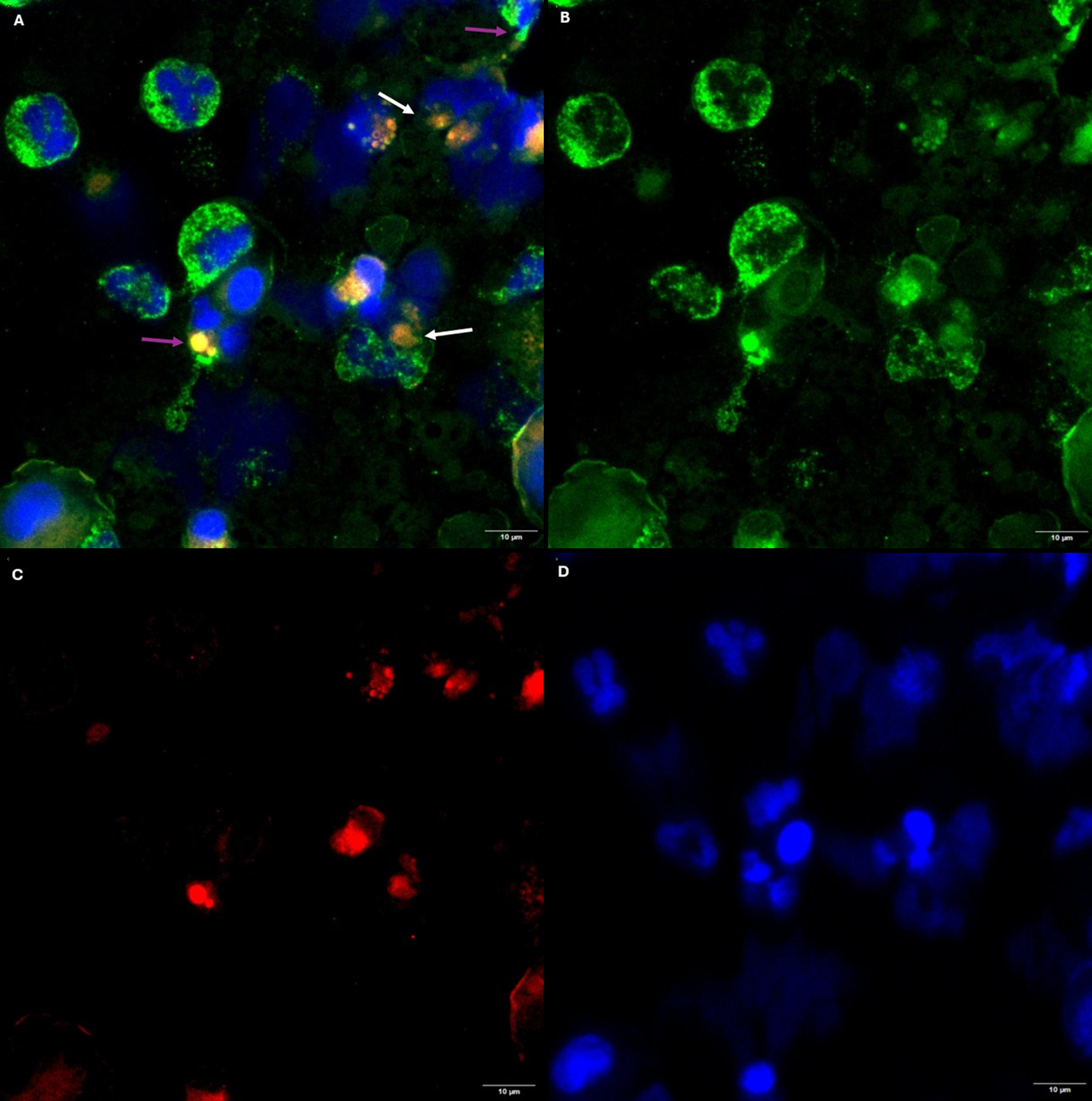

Supplement: SUPPLEMENTARY FIGURE S1 — Illustrative confocal microscopy pictures (objective 40x) of BALF neutrophils in bronchoalveolar lavage fluid of a dog with bronchiectasis (A). Activated neutrophils showing extracellular myeloperoxidase (MPO; green; B)- and citrullinated histones 3 (Cit-H3; red; C)-positive structures, consistent with ongoing NETs formation, are indicated by purple arrows on A. The white arrows indicate presence of intracellular MPO and Cit-H3-positive structures on A. DAPI (4′,6-diamidino-2-phenylindole) is a fluorescent dye used to label DNA (D). The scale bar indicates a length of 10 μm. [file Image_1.png]
