# Supplementary material for: Increased neutrophil extracellular traps formation in the bronchoalveolar lavage fluid of dogs with bronchiectasis
Source: Front Vet Sci. 2026 Mar 18;13:1786801. doi: 10.3389/fvets.2026.1786801 (PMC13040547; doi:10.3389/fvets.2026.1786801)
Supplement: Supplementary file 2 [file Table_1.docx]

Supplementary Table 1: Spearman correlation coefficients between MPO-DNA complexes and other variables for all dogs

| **Variables** | **Correlation** | **p-value** |
| --- | --- | --- |
| Age (years) | 0.10 | 0.49 |
| Weight (kg) | 0.15 | 0.30 |
| Duration of clinical signs (months) | 0.36 | 0.014* |
| TCC (cells/µl) | 0.38 | 0.0078* |
| Macrophage (%) | -0.40 | 0.0052* |
| Neutrophil (%) | 0.33 | 0.024* |
| Lymphocyte (%) | -0.11 | 0.45 |
| Eosinophil (%) | 0.15 | 0.32 |
| ANC (cells/µl) | 0.37 | 0.0098* |
| cfDNA (µg/ml) | 0.36 | 0.017* |

* Indicates a statistically significant correlation

TCC = Total cell count per µl; ANC = absolute neutrophil count; cfDNA = cell-free DNA
